# Somatic embryogenesis from seeds in a broad range of *Vitis vinifera* L. varieties: rescue of true-to-type virus-free plants

**DOI:** 10.1186/s12870-017-1159-3

**Published:** 2017-11-29

**Authors:** Tània San Pedro, Najet Gammoudi, Rosa Peiró, Antonio Olmos, Carmina Gisbert

**Affiliations:** 1 Instituto Valenciano de Investigaciones Agrarias (IVIA), Centro de Protección Vegetal y Biotecnología, Carretera de Moncada a Náquera km 4.5, 46113 Moncada, Spain; 2 Arid and Oases Cropping Laboratory, Arid Lands Institute (IRA), 4119 Medenine, Tunisia; 3 Instituto de Conservación y Mejora de la Agrodiversidad Valenciana (COMAV), Universitat Politècnica de València, Camino de Vera, 14, 46022 Valencia, Spain

**Keywords:** Grapevine, Direct and indirect embryogenesis, Microsatellites, TDZ, Sanitation

## Abstract

**Background:**

Somatic embryogenesis is the preferred method for cell to plant regeneration in *Vitis vinifera* L. However, low frequencies of plant embryo conversion are commonly found. In a previous work we obtained from cut-seeds of a grapevine infected with the *Grapevine leafroll associated viruses 1* and *3* (GLRaV-1 and GLRaV-3), high rates of direct regeneration, embryo plant conversion and sanitation. The aim of this study is to evaluate the usefulness of this procedure for regeneration of other grapevine varieties which include some infected with one to three common grapevine viruses (GLRaV-3, *Grapevine fanleaf virus* (GFLV) and *Grapevine fleck virus* (GFkV)). As grapevine is highly heterozygous, it was necessary to select from among the virus-free plants those that regenerated from mother tissues around the embryo, (true-to-type).

**Results:**

Somatic embryogenesis and plant regeneration were achieved in a first experiment, using cut-seeds from the 14 grapevine varieties Airén, Cabernet Franc, Cabernet Sauvignon, Mencía, Merlot, Monastrell, Petit Verdot, Pinot Blanc (infected by GFLV and GFkV), Pinot Gris, Pinot Meunier, Pinot Noir, Syrah, Tempranillo (infected by GFLV), and Verdil. All regenerated plants were confirmed to be free of GFkV whereas at least 68% sanitation was obtained for GFLV. The SSR profiles of the virus-free plants showed, in both varieties, around 10% regeneration from mother tissue (the same genetic make-up as the mother plant). In a second experiment, this procedure was used to sanitize the varieties Cabernet Franc, Godello, Merlot and Valencí Blanc infected by GLRaV-3, GFkV and/or GFLV.

**Conclusions:**

Cut-seeds can be used as explants for embryogenesis induction and plant conversion in a broad range of grapevine varieties. The high regeneration rates obtained with this procedure facilitate the posterior selection of true-to-type virus-free plants. A sanitation rate of 100% was obtained for GFkV as this virus is not seed-transmitted. However, the presence of GLRaV-3 and GFLV in some of the regenerated plants showed that both viruses are seed-transmitted. The regeneration of true-to-type virus-free plants from all infected varieties indicates that this methodology may represent an alternative procedure for virus cleaning in grapevine.

**Electronic supplementary material:**

The online version of this article (10.1186/s12870-017-1159-3) contains supplementary material, which is available to authorized users.

## Background

Virus infections are commonly found under field conditions in grapevine worldwide and multiple infections are routinely detected. Pathogenic agents, including 65 viruses, five viroids and eight phytoplasmas have been detected in a large number of infected grapevines [1]. Among these viruses, the most important are *Grapevine fanleaf virus* (GFLV), *Arabis mosaic virus* (ArMV), *Grapevine fleck virus* (GFkV), *Grapevine leafroll associated virus* (GLRaV) species which cause the ‘grapevine leafroll disease’ (GLD), and the rugose wood (RW) complex. Virus infections cause large economic losses in grapevine because of reductions in plant vigour, yield and fruit quality [2]. The long-distance spread of grapevine viruses occurs primarily by the propagation of infected plant material. Therefore, to produce certified material, plants free of the most dangerous viral pathogens are needed. It is essential the selection of virus-free plants or the sanitation of virus-infected plants to produce certified material and conserve germplasm for future necessities. Some viruses are particularly recalcitrant to elimination, so therefore different approaches has been tested for virus sanitation [3]: meristem culture [4, 5]; meristem culture combined with thermotherapy [6, 7], chemotherapy [8, 9], and somatic embryogenesis [3, 10–12].

Somatic embryogenesis consists in the induction of somatic embryos from cells of the explants cultured in vitro and is the preferred method for cell to plant regeneration in *Vitis vinifera* L. and its intraspecific or interspecific hybrids. It has been used for micropropagation [13, 14], generation of transgenic plants [15] and virus sanitation [3, 12]. Even though many studies have been carried out on embryogenesis in grapevine, the standardization of the conditions for embryogenesis induction and in vitro plant regeneration is still an empirical process because it depends on the genotype, type of explant, composition of the culture medium, physiological status of the donor plant, and culture conditions [16–18]. The most limiting factor in grapevine regeneration through somatic embryogenesis is the low rate of embryo to plant conversion, that is, a high percentage of embryos are not able to develop into normal plants [19–21]. In the work of Peiró et al. [12], additionally to plant sanitation, direct somatic embryogenesis (without callus formation) was achieved with high rates of embryo plant-conversion (starting from cut seeds). The time required to recover plants was also shortened with respect to other protocols.

In the present work, we report the results of two experiments aimed to obtain somatic embryo plants (SE-plants) from a total of 16 grapevine varieties (Airén, Cabernet Franc, Cabernet Sauvignon, Mencía, Merlot, Monastrell, Petit Verdot, Pinot Blanc, Pinot Gris, Pinot Meunier, Pinot Noir, Syrah, Tempranillo, Verdil, Godello, and Valencí Blanc), and rescue virus-free plants from grapevine varieties infected with one, two or three viruses commonly found in grapevine (GFLV, GFkV and GLRaV-3). In the first experiment, the putative influence of thidiazuron (TDZ) and genotype was also studied on both, somatic embryogenesis induction and embryo to plant conversion. Among the SE-virus-free regenerated plants, those regenerated from mother tissue (true-to-type) were selected via SSR genotyping.

## Results

### Regeneration via embryogenesis in fourteen grapevine cultivars, two of them virus infected

#### Embryogenesis induction

**Fig. 1:**
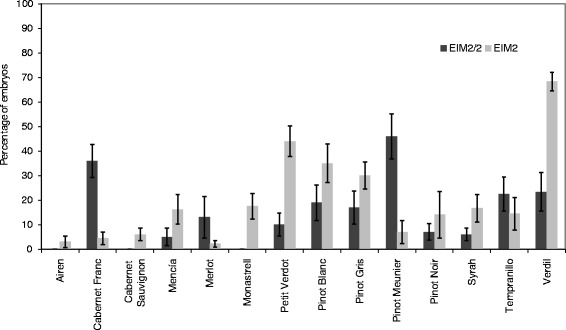
Percentage of germination in cut-seeds (from zygotic embryos) of the grapevine varieties Airén, Cabernet Franc, Cabernet Sauvignon, Mencía, Merlot, Monastrell, Petit Verdot, Pinot Blanc, Pinot Gris, Pinot Meunier, Pinot Noir, Syrah, Tempranillo, and Verdil after 1 month of culture on medium EIM2 (0.9 μM) and EIM2/2 (0.45 μM). Average of 10 plates per variety ± SE

**Fig. 2:**
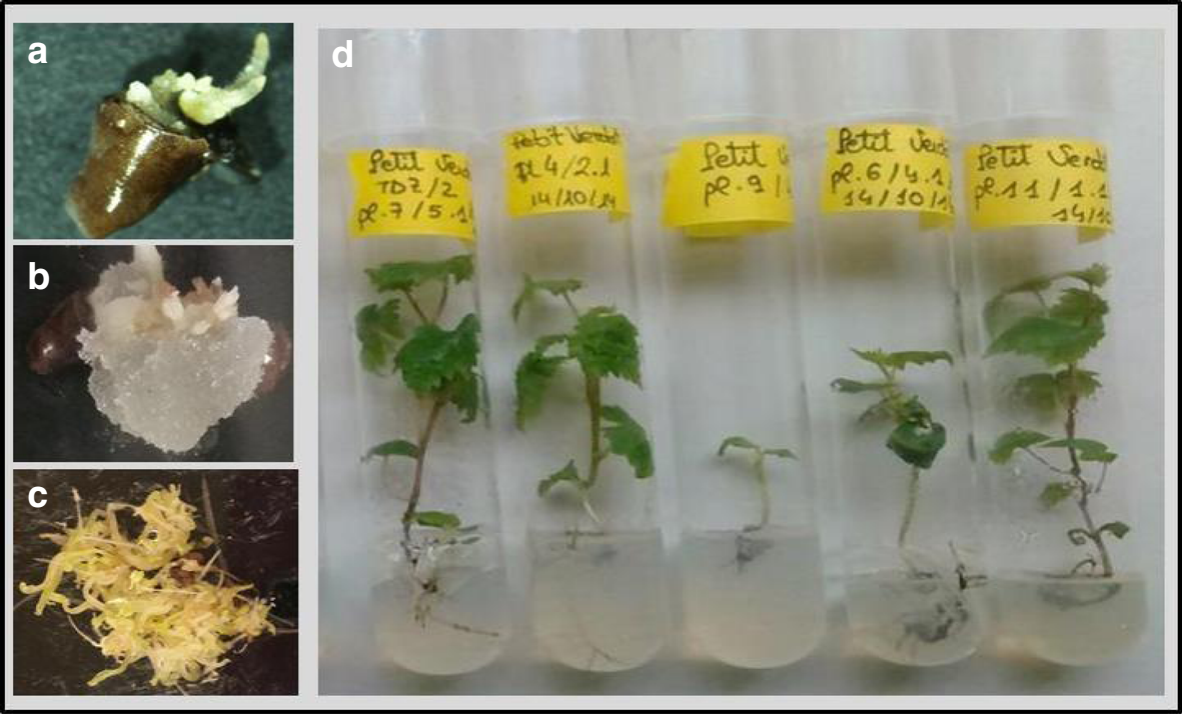
(**a-c**) Somatic embryogenesis (SE) induction after 2 months of culture of grapevine explants (cut seeds) on medium EIM2, and plant development: **a.** Direct SE in the variety Pinot Blanc. **b.** Indirect SE in an explant of the variety Monastrell. **c**. Somatic embryos in an explant of the variety Verdil. **d.** Rooted plants of variety Petit Verdot, 30 days after transferring somatic embryos (from the explants after 2 months of induction) to tubes with W medium. Among them, one plant has delayed development

In a first experiment, the regeneration ability was evaluated from cut-seeds of the grapevine varieties Airén, Cabernet Franc, Cabernet Sauvignon, Mencía, Merlot, Monastrell, Petit Verdot, Pinot Blanc (infected with GFLV and GFkV), Pinot Gris, Pinot Meunier, Pinot Noir, Syrah, Tempranillo (infected with GFLV) and Verdil, grown on medium containing TDZ at 0.90 (EIM2) or 0.45 μM (EIM2/2). After 1 month of culture, zygotic germination from cut-seeds was observed in some varieties and media conditions (Fig. 1). The germination percentage was estimated (ranging from 2.2%, for variety Merlot cultured on EIM2, to 68.3% for Verdil cultured on EIM2/2) and these plants were then removed. On average, germination was higher for seeds cultured on the medium with higher concentration of TDZ (EIM2) (22.35% vs 11.79%) and did not occur in Airén, Cabernet Sauvignon, and Monastrell cultured on medium EIM2/2. In non-cut seeds (sown as controls), germination was absent in both Murashige and Skoog basal medium (MS) as well as in EIM2. After 2 months of culture (T2), SE was observed in all the assayed varieties (Table 1). The percentage of embryogenic explants ranged from 18.50%, in Airén, to 65.46%, in Verdil and it was mainly achieved via direct embryogenesis (without callus formation) which was observed in at least 90.0% of the explants of the varieties Cabernet Franc, Mencía, Petit Verdot, Pinot Blanc, Pinot Gris, Pinot Meunier, Pinot Noir, and Verdil (Table 1; Fig. 2a). On the contrary, Airén and Monastrell showed great callus formation, that is indirect embryogenesis (Table 1; Fig. 2b). After three (T3) and 4 months of culture (T4), the somatic embryogenesis percentage (E) had increased, decreased (new embryos were not observed in explants which had responded in a previous period), or been maintained at similar rates - with respect to the previous period - depending on the cultivar (Table 1). There was no effect of the TDZ concentration on direct embryogenesis (DE); both concentrations were able to induce regeneration from cut seeds at a similar extent. Concerning total embryogenesis, no effect of TDZ was observed at T3 and T4, except at T2, when a higher percentage of total embryogenesis was observed in EIM2 with respect to EIM2/2 (48.30% vs 40.29%, respectively. Data not shown).

**Table 1:** Percentages of total embryogenic explants (E, %) and embryogenic explants obtained via direct embryogenesis (DE, %) after two (T2), three (T3), and four (T4) months of culture. Starting explants consist of 100 seeds distributed a rate of 10 seeds per Petri dish

Cultivar	T2		T3		T4	
	E	DE	E	DE	E	DE
Airen	18.50 j	0.50 h	31.50 fg	1.50 g	13.00 g	1.00 f
Cabernet Franc	51.26 bcde	51.09 abc	60.50 abc	59.91 a	64.66 a	64.65 a
Cabernet Sauvignon	28.99 hij	23.79 fg	51.15 cd	50.17 abc	46.26 b	44.06 b
Mencía	43.77 defg	40.09 cde	52.17 cd	48.71 abcd	28.73 def	28.71 cde
Merlot	38.10 ghi	35.30 e	54.71 bcd	50.44 abc	37.82 bcd	37.81 bc
Monastrell	42.00 efg	4.75 h	46.44 de	7.55 fg	42.85 bc	25.20 de
Petit Verdot	55.50 abc	49.00 bcd	64.00 ab	56.00 ab	71.00 a	70.50 a
Pinot Blanc	50.00 bcdef	50.00 abc	30.50 fg	30.50 e	22.00 fg	22.00 e
Pinot Gris	54.94 abcd	54.78 ab	45.24 de	45.17 bcd	25.71 ef	25.70 de
Pinot Meunier	60.50 ab	60.50 a	48.50 de	48.50 bcd	44.50 bc	44.50 b
Pinot Noir	39.00 fgh	39.00 de	39.00 ef	39.00 de	34.50 cde	34.00 bcd
Syrah	27.57 ij	16.88 g	26.29 g	15.70 f	26.76 def	19.39 e
Tempranillo	44.47 cdefg	28.77 ef	48.26 de	39.50 cde	48.90 b	44.21 b
Verdil	65.46 a	60.83 a	70.58 a	56.34 ab	49.71 b	42.34 b
ANOVA Cultivar Culture medium Interaction	* * NS	* NS NS	* NS NS	* NS NS	* NS NS	* NS NS

Different lower case letters within a column indicate significantly different values (*P* value < 0.05)

*NS*: *P* value >0.05

*: *P* value <0.05

Taking into account the data of T2, T3, and T4, the most responsive cultivars were Verdil, Petit Verdot, and Cabernet Franc, followed by Pinot Meunier, which had, on average, around 60.0% of embryogenic explants. The less responsive varieties were Airén and Syrah, with maximum E values of 31.5% and 27.57% at T3 and T2, respectively. As Pinot Blanc and Tempranillo were virus-infected, the percentage of regeneration could be different if seeds from uninfected plants were used. However, around 40% regeneration was obtained in both varieties which suggests that viruses did not inhibit the regeneration response.

Effects of the cultivars and media were found when the number of embryos per explant was analyzed. Besides, the interaction between the cultivars and media was also significant. Few differences were observed among cultivars in the number of embryos per explant, mainly due to variability in the number of embryos per explant and consequently a high error in the estimation. In addition, whereas in medium EIM2 embryo production was similar at T2 and T3, fewer embryos were obtained in EIM2/2 at T3 with respect to T2. Regarding the interaction, the best responding cultivar was Verdil, which had around 10 embryos per explant when cultured on EIM2 (Table 2) and had explants with more than 20 embryos (Fig. 2c). In this cultivar, the amount of embryos per explant was halved when the TDZ concentration in the medium was reduced (an average of 5.11% in EIM2/2 vs 10.54% in EIM2). A similar result was found in Petit Verdot. Considering the percentages of embryogenic calli, the maintenance of embryogenic ability over 4 months, and the average number of embryos per explant at T2 and T3, the most responsive genotypes were Verdil and Petit Verdot, followed by Cabernet Franc and Pinot Meunier.

**Table 2:** Number of embryos per explant (NE) for each cultivar after two (T2) and three (T3) months of culture, and the average values. Starting explants consist of 100 seeds distributed a rate of 10 seeds per Petri dish

Cultivar	Medium	NE at T2	NE at T3	NE/explant
Airen	EIM2	1.70 ± 0.53 b	1.02 ± 0.30 d	1.36 ± 0.26 cd
	EIM2/2	0.83 ± 0.60 b	1.21 ± 0.10 cd	1.02 ± 0.41 d
Cabernet Franc	EIM2	1.24 ± 0.62 b	1.61 ± 0.57 cd	1.43 ± 0.58 cd
	EIM2/2	2.51 ± 0.25 b	3.16 ± 0.21 bc	2.84 ± 0.24 cd
Cabernet Sauvignon	EIM2	1.39 ± 0.39 b	1.51 ± 0.14 cd	1.45 ± 0.23 cd
	EIM2/2	1.14 ± 0.41 b	1.25 ± 0.23 cd	1.19 ± 0.27 cd
Mencía	EIM2	1.34 ± 0.38 b	1.15 ± 0.15 cd	1.24 ± 0.22 cd
	EIM2/2	2.03 ± 0.25 b	1.12 ± 0.25 cd	1.57 ± 0.25 cd
Merlot	EIM2	1.57 ± 0.49 b	1.10 ± 0.65 cd	1.34 ± 0.51 cd
	EIM2/2	2.19 ± 0.34 b	1.99 ± 0.35 cd	2.09 ± 0.32 cd
Monastrell	EIM2	2.75 ± 0.39 b	2.71 ± 0.34 bcd	2.73 ± 0.24 cd
	EIM2/2	2.17 ± 0.58 b	1.25 ± 0.64 cd	1.71 ± 0.61 cd
Petit Verdot	EIM2	2.14 ± 0.27 b	4.15 ± 0.47 b	3.15 ± 0.41 bc
	EIM2/2	1.32 ± 0.40 b	1.79 ± 0.63 cd	1.55 ± 0.55 cd
Pinot Blanc	EIM2	2.05 ± 0.19 b	1.40 ± 0.59 cd	1.73 ± 0.32 cd
	EIM2/2	1.19 ± 0.35 b	2.24 ± 0.30 bcd	1.72 ± 0.32 cd
Pinot Gris	EIM2	1.35 ± 0.12 b	1.12 ± 0.14 cd	1.24 ± 0.12 cd
	EIM2/2	1.20 ± 0.28 b	1.07 ± 0.20 d	1.14 ± 0.26 cd
Pinot Meunier	EIM2	1.78 ± 0.31 b	1.81 ± 0.61 cd	1.79 ± 0.43 cd
	EIM2/2	1.15 ± 0.38 b	1.83 ± 0.66 cd	1.49 ± 0.42 cd
Pinot Noir	EIM2	1.35 ± 0.26 b	1.42 ± 0.35 cd	1.39 ± 0.29 cd
	EIM2/2	1.19 ± 0.22 b	1.36 ± 0.58 cd	1.27 ± 0.36 cd
Syrah	EIM2	1.00 ± 0.25 b	0.94 ± 0.27 d	0.97 ± 0.21 d
	EIM2/2	1.21 ± 0.13 b	1.35 ± 0.45 cd	1.28 ± 0.19 cd
Tempranillo	EIM2	2.20 ± 0.42 b	1.23 ± 0.36 cd	1.71 ± 0.31 cd
	EIM2/2	2.12 ± 0.49 b	2.15 ± 0.44 bcd	2.14 ± 0.36 cd
Verdil	EIM2	10.77 ± 0.55 a	10.32 ± 0.25 a	10.54 ± 0.33 a
	EIM2/2	8.72 ± 0.58 a	1.50 ± 0.25 cd	5.11 ± 0.53 b
ANOVA	Cultivar Culture medium Interaction	*** * ***	*** * ***	*** * ***

Different lower case letters within a column indicate significantly different values (*P* value < 0.05)

*: *P* value <0.05

***: *P* value <0.001

### Germination of SE: Embryo conversion

In the present work, the induced somatic embryos were able to germinate directly in the induction medium (Fig. 2c). To study the development of somatic embryos, 20 germinated embryos per cultivar (from several explants which showed direct embryogenesis) at T2 and T3 were transferred to individual tubes for growth (Fig. 2d). Different parameters related to plant embryo conversion were measured 20 and 40 days later. After 20 days of culture, a low percentage of embryos with normal aspect were observed in the variety Cabernet Sauvignon. Around 90% of plantlets showed white cotyledons, presence of one cotyledon, or fused cotyledons. On the contrary, normal appearance was observed in all plantlets of Pinot Gris and Pinot Meunier. For the rest of the varieties, normal plantlets were in the range of 50–90% (Table 3). In order to compare the development of embryos, we used an index for shoot development (DI) - which takes into account the percentage of plantlets with leaves and the mean number of leaves per plantlet having leaves - and another index for rooting (RI), based on visual root development (Additional file 1: Table S1). The highest and the lowest DI values correspond to the cultivars Pinot Meunier and Cabernet Sauvignon, respectively. The highest RI value was noted for the Verdil cultivar, differences among the other grapevine varieties were scarce. In Fig. 2d, we can see rooted plantlets of the variety Petit Verdot, at different development stages, after 30 days of culture. Along the culture period, some plants that seemed abnormal at 20 days of culture had developed into normal plants 40 days after. This occurred in all the cultivars with the exception of Syrah and Airén, which had a similar embryo conversion (EC) values at both periods. After 40 days of culture, EC was: 100% in Cabernet Franc, Pinot Gris, Pinot Meunier, Tempranillo, and Verdil; 80–95% in Merlot, Pinot Blanc, and Pinot Noir; 50–75% in Airén, Monastrell, Petit Verdot, and Syrah; and around 30% in Cabernet Sauvignon (Table 3). Whereas at 20 days of culture, differences in DI were observed between Pinot Meunier and the rest of the genotypes, at 40 days of culture there were no differences in DI among this variety and Tempranillo, Merlot, and Cabernet Franc. Similar to the results showed above, Pinot Meunier was more developed than the other Pinot cultivars (Additional file 1: Table S1).

**Table 3:** Percentages of embryos with normal cotyledons (EC: embryo conversion, %). As starting explants, 20 somatic embryos from direct embryogenesis events were sown in individual tubes

Cultivar	EC	EC
	20 days	40 days
Airén	52.09 bc	54.77 cd
Cabernet Franc	80.00 ab	100.00 a
Cabernet Sauvignon	11.11 c	29.37 d
Merlot	75.00 ab	80.00 abc
Monastrell	53.46 bc	67.27 abc
Petit Verdot	66.67 ab	75.00 abc
Pinot Blanc	67.78 ab	94.44 ab
Pinot Gris	100.00 a	100.00 a
Pinot Meunier	100.00 a	100.00 a
Pinot Noir	52.50 bc	81.25 abc
Syrah	61.12 ab	61.12 bcd
Tempranillo	88.89 ab	100.00 a
Verdil	78.75 ab	100.00 a

Different lower case letters within a column indicate significantly different values (*P* value < 0.05)

In addition, flow cytometry analysis demonstrated that all the plantlets obtained by this procedure had the same DNA content as the mother plants, meaning they were diploid.

### Rescue of virus-free plants and SSR analysis

The results of real time RT-PCR to detect virus presence in SE-plants from Tempranillo and Pinot Blanc are shown in Additional file 2: Table S2. Of 16 Tempranillo SE-plants, 11 were GFLV-free, representing 69% sanitation. With respect to Pinot Blanc, all of the eight plants analyzed were free of GFkV (100% of virus-free plants) and one SE-plant remained infected with GFLV (88% sanitized). The SSR profile by analyzing six SSRs (Additional file 2: Table S2) indicates that two SE virus-free plants of Tempranillo and one SE virus-free plant of Pinot Blanc were true-to-type. Therefore, around 10% of the plants were sanitized and displayed the same allele profile as the mother plant.

### Virus sanitation of the virus-infected grapevines varieties Cabernet Franc, Merlot, Godello, and Valencí Blanc

Embryogenesis was induced in cut-seeds cultured on EIM2 (Table 4) in explants from all four cultivars each infected with two viruses (Table 5). Mean rates of regeneration indicated that Valencí Blanc was the lowest responding variety of those assayed in this experiment. As occurred in the previous assay, direct embryogenesis was found with high rates in Cabernet Franc and Merlot whereas indirect embryogenesis predominated in Valencí Blanc and Godello (Table 4). After germination of SE, SE-plants were analyzed in order to assess ploidy and to detect the putative presence of the viruses detected in the mother plants. All SE-plants showed similar DNA-ploidy pattern to mother plants (data not shown) but different sanitation rates were obtained depending on viruses and the variety. All the analyzed SE-plants were GFkV-free. However, the presence of GLRaV-3 was found in a Merlot SE-plant, and GFLV in three Godello and one Merlot SE-plants. Therefore, 100% of sanitation was obtained in Cabernet Franc and Valencí Blanc, and ca 80% in Merlot and Godello. These results together with those obtained in the first experiment suggest that the most difficult virus for sanitation among those analyzed is GFLV. The detection of GLRaV-3 and GFLV in SE-plants confirmed the presence of these viruses in the seeds used as starting explants.

**Table 4:** Percentages of total embryogenic explants (E, %) and embryogenic explants obtained via direct embryogenesis (DE, %) after two (T2), three (T3), and four (T4) months of culture, and their average. Starting explants consist of 100 seeds distributed a rate of 10 seeds per Petri dish

Cultivar	T2		T3		T4		Average	
	E	DE	E	DE	E	DE	E	DE
Cabernet Franc	60.00 ^b^	57.78^c^	68.89^b^	63.33^b^	73.33^c^	61.11^c^	67.40^b^	60.74^d^
Godello	55.00^c^	11.00^a^	77.00^c^	11.00^a^	70.00^c^	9.00^a^	67.33^b^	10.33^b^
Merlot	54.44^b^	41.11^b^	53.33^b^	51.11^b^	54.44^b^	40.00^b^	54.07^b^	44.07^c^
Valencí Blanc	13.00^a^	2.00^a^	17.00^a^	1.00^a^	29.00^a^	5.00^a^	19.67^a^	2.67^a^

Different lower case letters within a column were significantly different (*P* value < 0.05)

In bold mother plants and SE plants with the same SSR profile than the mother plant

**Table 5:** Virus status of mother plant and plants from SE (somatic embryos) obtained for each cultivar. Percentages of sanitation and percentages of plants regenerated from mother tissue (true-to-type)

Cultivar	Virus status of mother plants	SE plants analyzed	N sanitized plants	% sanitation	SE plants that remain infected	Plants true-to-type^1^	% true-to-type/sanitized plants
Cabernet Franc	GLRaV-3 and GFkV	15	15	100	0	1	6.7
Godello	GLRaV-3 and GFLV	14	11	79	3 with GFLV	1	9.1
Merlot	GLRaV-3 and GFLV	10	8	80	1 with GLRaV-3 1 with GFLV	1	12.5
Valencí Blanc	GLRaV-3 and GFkV	10	10	100	0	1	10.0

Results of SSRs analysis in the regenerated plants (Additional file 3: Table S3) showed that at least one plant virus-free per cultivar was true-to-type. The percentage of true-to-type plants with respect to sanitized plant is on average around 10%, similarly to that found in the first assay. Therefore, this methodology permits the rescue of useful grapevine plants free of GFkV, GLRaV-3 and GFLV, even if mother plants are infected with all of these viruses.

## Discussion

The high rates of direct regeneration and plant conversion starting from cut seeds described in our previous work aiming to sanitize the cultivar Valencí Negre [12] led us to evaluate the procedure in other varieties to induce regeneration and estimate the efficiency in virus sanitation. This later implicated the analysis of the regenerated virus-free plants genotypes using SSRs (useful plant were those regenerated from mother tissue).

In the first experiment, seed germination (zygotic) after 1 month of culture occurred from some cut-seeds. This effect that was not observed by Peiró et al. [12] and may be consequence of the germination of undamaged embryos after cutting (germination was not observed in non-cut seeds cultured as controls). Despite germination was not the purpose of this work, this result could be interesting in breeding programs to reduce the intervals in the progenies evaluations as reported in grapevine by Ramming et al. [22].

Embryogenesis induction and direct regeneration as reported in Peiró et al. [12], was observed in all the 16 assayed varieties, with high rates in 12 of them. This kind of regeneration is convenient since it may reduce somaclonal variation [23–25]. The most common growth regulators used to induce embryogenesis in grapevine are dichlorophenoxyacetic acid (2,4-D) combined with 6-benzyladenine (6-BA) [16, 26] which generally produce high callus formation. The effect of the concentration of TDZ (reduced by half in EIM2/2) was also evaluated in this experiment to increase the possibilities of embryo induction and/or plant conversion. The concentration of the growth regulator is a key factor in both processes and TDZ, a powerful growth regulator, may interfere in shoot development in excess [27, 28]. The obtained results showed that a concentration of 0.9 μM of TDZ is adequate for embryogenesis induction and plant development in grapevine. However, similar or higher response was obtained in EIM2 medium with respect to EIM2/2 medium. Therefore, EIM2 medium was used for the second experiment where induction of embryogenesis and embryo to plant conversion was achieved in the grapevine varieties Cabernet Franc, Merlot, Godello, and Valencí Blanc.

The fact that high percentages of embryogenic explants were produced after only 2 months of culture from different parts of cut-seeds in all the varieties indicates that regarding embryogenesis induction this protocol is faster than others, where five [3, 10, 29] or 7 months [18] are required. In addition, the lowest E values obtained in the present work (in Airén or Valencí Blanc; E > 19%) were higher than those reported in other works that used anthers and ovaries as starting explants. For instance, Martinelli et al. [30] reported 2.0% of embryogenic calli in anther and 14.0% in ovary cultures in the Chardonnay cultivar, while E values from 2 to 7% were reported by Oláh et al. [31] - who used anthers of seven interspecific hybrids (*V. berlandieri* x *V. rupestris* or *V. riparia*). The percentages of embryogenesis obtained in the present work were also higher than those reported from stamens and pistils by Prado et al. [18], who achieved an E value of 23.0% in the Mencía cultivar in the best treatment, and Dhekney et al. [17], who reported E values in the range from 0.4 to 35.0% for the best treatments in Cabernet Franc, Cabernet Sauvignon, Merlot, Pinot Gris, Pinot Noir, and Syrah explants. On the other hand, Oláh et al. [32] did not obtained embryogenesis in anthers from the varieties Cabernet Sauvignon, Merlot, Pinot Gris, or Pinot Noir when they used medium containing TDZ (0.22 μM) combined with 2,4-D (4.97 μM) and low efficiencies (E < 5%) were achieved in a medium with 2,4-D (5 μM) and 6-BA (0.44 μM). In the present work, an E value of 27% was achieved in the variety Syrah and E values in a range from 40 to 59% in Cabernet Franc, Cabernet Sauvignon, Mencía, Merlot, Pinot Gris, and Pinot Noir. Besides, this is the first report of successful embryogenesis induction for the varieties Airén, Monastrell, Petit Verdot, Pinot Blanc, Verdil, Godello, and Valencí Blanc. Among the four Pinot varieties, Pinot Meunier had a higher response than Pinot Blanc, Pinot Gris, and Pinot Noir regarding both E and DE. Data from SSR analysis showed higher variability in this cultivar with respect to the other Pinot varieties, which are considered as mutations at the berry colour locus of the variety Pinot Noir [33, 34].

The final step of the embryogenesis procedure is the conversion of embryos into plants. Few reports have focused on this aspect even though a high percentage of grapevine embryos are not able to develop into normal plants [19–21]. In our work, germination of SE occurred directly in the induction medium and embryos were transferred to growth regulator-free medium to follow their development. Noteworthy, several other works reported the requirement of additional labour-intensive steps to go from embryo to plant. For instance, Martinelli et al. [30] first separated clusters of embryos from the embryogenic calli and placed them in a liquid medium; then, the embryos - with the radicals facing downward - were transferred to a medium with 6-BA and IBA or to hormone-free medium for germination. López-Pérez et al. [26] transferred table-grape embryos to a medium with indoleacetic acid, gibberellic acid, and activated charcoal (AC) and then, after germination took place, transferred them to a half-strength MS medium. Comparing to data reported in the literature, the percentages of EC obtained in the present work were high (in average 80.25% after 40 days of culture). EC values around 48%, 55%, and 73% were reported by Dhekney et al. [17] in the varieties Cabernet Franc, Syrah and Merlot, respectively, and an EC of 13.6% was reported for Cabernet Sauvignon by Ben Amar et al. [21]. In varieties different from those used in this work, Goebel-Tourand et al. [19] failed to exceed 20% conversion whereas for López-Pérez et al. [20] conversion ranged from 42.7% to 63.8%. The fact that all the tested varieties in this study produced plants that were well rooted and developed indicates that the growth medium used was good enough for rooting all of them.

Virus sanitation by induction of embryos from stamens or pistils was reported in grapevine to cure plants from GFLV [29], GLRaV [3, 11] or ArMV [10] viruses. In the present work, SE-free plants were obtained from plants infected with at least one of the following viruses: GFkV, GFLV or GLRaV-3. The presence of GFkV was not observed in any of the analyzed SE-plants. This was expected as GFkV was reported as not seed transmitted [35]. However, in our study GFLV and GLRaV-3 were detected in some SE-derived plants by RT-PCR indicating that these viruses were in the seed explants. Whereas GFLV was described to be transmitted by seeds in grapevine [36], to the best of our knowledge this is not reported for GLRaV-3 [35, 37]. Despite this result, the sanitation percentages obtained in this work were high (68–100%) for all tested virus and varieties. This result confirms the usefulness of embryogenesis for virus sanitation and the use of seed as starting explants. Sexual embryos are also common explants for somatic induction in other woody species like oak [38], fraser fir [39] or elm [40]. Since grapevine is highly heterozygous, it is propagated vegetatively to keep the same genetic make-up as the parental material. For this reason, we had to select from among the SE-plants, those regenerated from the coat tissue (mother tissue). In grapevine, egg and central embryo sac cells each fuse with sperm cells giving rise to embryo and endosperm development, respectively, whereas seed coats develop from ovule sporophytic tissue [41]. With this aim, the SSRs developed for identification of grape cultivars [42] and recommended by the OIV were used in the first experiment showing that ca 10% of the SE virus-free plant, for each cultivar, were true-to-type. In the second experiment, we increased the number of SSRs (from six to nine), obtaining similar rates of true-to-type sanitized-plants. The use of SSR is common to identify grapevine varieties as well as determine relationships [43, 44]. In conclusion, around 10% of SE plants were suitable for germplasm storage and/or plant multiplication (sanitized and true-to-type). We cannot compare our efficiency respect to that obtained in works that used ovaries or anthers for grapevine embryogenesis induction and virus sanitation [3, 10, 11, 29, 45] because the true-to-type character of SE-plants was not analysed in any of them. In comparison with other sanitation procedures, like meristem culture, the procedure described in the presented study offers the possibility to collect many starting explants which are easier to manipulate than the meristem. Despite meristem culture is the preferred technology for virus cleansing, embryogenesis may be an alternative to consider. Very small meristems are needed for efficient virus cleansing but sometimes the rescue of virus-free plants is not easy. High amount of meristem from previously in vitro micropropagated plants are also needed. Although good results have been obtained with chemotherapy for some virus and specific varieties, its success depends on the virus, the toxicity produced by anti-viral chemicals, and the variety [8, 46, 47].

## Conclusions

The results obtained in two independent experiments showed that the culture of cut-seeds on media containing TDZ is appropriated for embryogenesis induction and plant conversion in a broad sense of grapevine varieties. Cut seeds have the advantage - with respect to other commonly used explants (ovaries and anthers) - that they are easy to collect and use. In comparison with other protocols of grapevine embryogenesis, this procedure reduces the time needed to regenerate plants. In addition, it was confirmed using molecular markers that some embryos were regenerated from mother tissue, which indicates the suitability of this procedure to be applied for different biotechnological purposes such as virus sanitation validated in this work. The high rates of sanitized regenerated plants facilitate the selection of true-to-type ones. In this work, virus-free and true-to-type plants of Tempranillo, Pinot Blanc, Cabernet Franc, Godello, Merlot and Valencí Blanc were obtained from grapevines with GLRaV-3, GFLV and/or GFkV in single or multiple infection. These results open up the possibility of using this technique for the sanitation of grapevine plants infected with other viruses. With respect to virus transmission, GFkV was not found in any of the analyzed plants which was expected because this virus is not transmitted by seed. However, the presence of GLRaV-3 and GFLV in some of the regenerated plants pointed out that both viruses are seed-transmitted.

## Methods

### First experiment: Embryogenesis induction in fourteen grapevine varieties, two of them virus infected

#### Plant material, virus detection and somatic embryogenesis

Grapes of the cultivars Airén, Cabernet Franc, Cabernet Sauvignon, Mencía, Merlot, Monastrell, Petit Verdot, Pinot Blanc, Pinot Gris, Pinot Meunier, Pinot Noir, Syrah, Tempranillo, and Verdil were collected in the summer of 2014 in an experimental field ‘Campo de experiencias El Rebollar’ belonging to the ‘Instituto Tecnológico de Viticultura y Enología’ sited at Requena (Valencia, Spain). Asymptomatic leaves from all the varieties were used to analyze putative virus infection through the methodology described by López-Fabuel et al. [48]. Briefly, extracts were prepared from leaves 1/20 *w*/*v* in PBS (Phosphate-Buffered Saline) buffer, pH 7.2, supplemented with 0.2% diethyldithiocarbamic acid (DIECA), and 2% polyvinylpyrrolidone-10 (PVP-10) in individual plastic bags with a heavy net (Plant Print Diagnostics, Valencia, Spain). Total RNA was extracted from 200 μl of crude extract using an Ultraclean Plant RNA isolation kit (Mobio, Carlsbad, CA, USA) following the manufacturer’s instructions. The Real-time multiplex RT-PCR was performed for the simultaneous detection of ArMV, GFLV, GFkV, GLRaV-1, and GLRaV-3 using a StepOne Plus thermocycler (Applied Biosystems) and a reaction mixture containing 1× AgPath-ID One-step RT-PCR buffer (Ambion) and 1.5× AgPath-ID One-step RT-PCR enzyme mix (Ambion); 5 μL of sample; 400 nM of GFLV, ArMV, GFkV, and GLRaV-1 primers; 800 nM of GLRaV-3 primers; and 200 nM of each probe (Additional file 4: Table S4). The amplification protocol consisted of an RT step at 45 °C for 10 min and a denaturation step at 95 °C for 10 min, followed by 45 cycles of amplification (95 °C, 15 s; 50 °C, 15 s; and 60 °C, 60 s). As positive controls viral isolates maintained at the Instituto Valenciano de Investigaciones Agrarias (IVIA) were included. When amplification was observed for a specific virus, it was confirmed by real-time uniplex RT-PCR with the corresponding primers.

Immature seeds were extracted by hand from grapes with a mean weight from 0.65 to 0.85 g (6.0 to 7.0 mm long × 3.0 to 3.5 mm wide), corresponding to stage 33 in Pierce and Coombe [49]. The seeds were surface sterilized and cut transversely, before subculturing them on embryogenesis induction medium 2 (EIM2) [12] - that contains salts and vitamins of the McCown Woody plant medium (DUCHEFA, The Netherlands), 4% sucrose, 0.01% polyvinylpyrrolidone-10 (PVP-10), 0.75% plant agar, 0.2% AC, and 0.90 μM TDZ (sterilized by filtration and added to the sterile medium) - or on EIM2/2 medium (equal composition of EIM2 except for TDZ: 0.45 μM). The pH of both media was adjusted to 5.8 before sterilization at 121 °C for 20 min. For each genotype and medium, 10 cut seeds were cultured per Petri dish and 10 repetitions (Petri dishes) per cultivar and treatment were evaluated. While culturing, some plates were removed when contamination was observed and at least eight Petri dishes per culture medium and genotype were used. A sample of non-cut seeds was also cultured on MS and EIM2. The seeds were cultured in darkness for 4 months, checking the plates monthly. After 1 month of culture, the percentage of seed germination (G) was noted and plantlets were removed from the seed. The percentage of seeds with embryogenic explants with DE, and number of embryos per responding explant (NE) were annotated after two, three, and 4 months of culture (T2, T3, and T4, respectively).

At T2 and T3, for each cultivar, 20 embryos which were initiating germination were transferred to tubes (one embryo/tube) with W medium [50] that contains Lloyd and McCown Woody plant salts, 2% sucrose, 0.01% PVP-10, 0.75% plant agar; supplemented with 1 μM indolbutyric acid (IBA) for growth under standard conditions: incubation in a growth chamber at 26 ± 2 °C under a 16 h photoperiod with cool white light provided by Sylvania cool white F37 T8/CW fluorescent lamps (90 μmol m^−2^ s^−1^). Embryos from each variety were selected from different seeds with direct embryogenesis, although sometimes 2 or 3 embryos per explant were used. Twenty and 40 days after transferring the embryos to tubes, the percentages of normal plantlets (EC) were noted. An index for plant development (DI) was calculated by multiplying the percentage of plantlets with leaves (expressed as a decimal) by the mean number of leaves per plant with leaves. A qualitative index (RI: 1–3, 1: small roots; 2: main root 1.0–1.5 cm or the presence of some secondary roots, 3: main root >1.5 cm and/or with many secondary roots) was used for scoring the rooting.

### Ploidy, virus and SSR analysis of SE-plantlets

DNA ploidy in at least eight SE-plants per variety was analyzed by flow cytometry like described by Gisbert [51] using as starting material for nucleus extraction leaves from the plants cultured in vitro.

Total RNA was extracted from 16 young leaves of SE-plantlets derived from the grapevine cultivar Tempranillo and eight young leaves of SE-plantlets from the cultivar Pinot Blanc. The Real-time uniplex RT-PCR was performed following the same methodology described previously in mother plants.

DNA was extracted from young leaves of SE-plantlets using DNA plant kit (Qiagen). DNA quality and quantity was assessed using gel electrophoresis and spectrophotometry. A multiplex PCR procedure was performed to amplify VVMD5, VVMD7, VVMD27, VVS2, VrZAG62 and VrZAG79 SSRs as described by This et al. [41]. Briefly, the forward primer of the SSR markers was labelled with one of the four fluorescent dyes, VVMD5 and VVMD27 used carboxy fluorescein (FAM), VVMD7 used carboxytetramethylrhodamine (TAMRA), VrZAG62 used hexachloro-6-carboxyfluorescein (HEX) and VVS2 and VrZAG79 used 6-carboxytetramethyl rhodamine (ROX). Multiplex PCR was carried out in a total volume 11 μl volume using 1.25 μL of commercial Master Mix PCR Multiplex (Takara Multiplex Hot Short PCR, Takara), 20–40 ng of genomic DNA and labeled multiplexed SSR primers (from 5.5 to 35 μM). The amplification was performed in an ABI 9700 thermocycler, and the amplification conditions were 95 °C for 14 min followed by 30 cycles of 95 °C for 30 s, 55 °C for 90 s, and 72 °C for 60 s, and a final extension of 72 °C for 30 min. Previously to PCR fragment size determination, multiplex PCR products were previsualized using gel electrophoresis. The electrophoresis was carried out on an ABI 3100 platform (Applied Biosystems, Foster City, CA, USA). For PCR fragment size determinations, 0.13 μl of an internal size standard (GeneSacn™ 500 LIZ, Applied Biosystems, Foster City, CA, USA) was mixed with 1 μl of PCR product and 10.87 μl formamide. The mixture was heated at 94 °C for 3 min and then cooled within icy water. The size of the SSR fragments was determined with the software packages GeneScan 3.7 (Applied Biosystems).

The microsatellite profile of SE plantlets was compared to microsatellite profile from Tempranillo and Pinot Blanc mother plant (source of seeds explants). Both varieties resulted virus-infected after the virus analysis.

### Second experiment: Sanitation of the virus-infected cultivars Cabernet Franc, Merlot, Godello, and Valencí Blanc

Leaves from the cultivars Cabernet Franc, Merlot, Godello, were collected in July of 2015 in the experimental field ‘El Rebollar’ (like in the first experiment) and those of Valencí Blanc in a particular garden at Penàguila (Alicante, Spain) and they were used to analyze virus presence following the same methodology described for the first experiment. Briefly, the sanitary statuses of mother plants were analyzed by real-time multiplex RT-PCR and were confirmed by real-time uniplex RT-PCR. Seeds were collected from the analyzed grapevine varieties and embryogenesis induction was performed by culture on medium EIM2 which contains TDZ at 0.9 μM. After 1 month of culture embryos that occasionally germinated were removed, and somatic embryos at T2, T3 and T4 were transferred to tubes with W medium for growth. Similarly to experiment 1, the ploidy analysis and the sanitary status of at least 10 SE-plants per cultivar were analyzed. In this experiment, SSRs VVMD25 (ROX), VVMD28 (HEX) and VVMD32 (FAM) were also included to increase the probability to assign the true-to-type. The PCR conditions were the same described above and the allele size determination was performed using the same software packages. The microsatellite profile of SE plantlets was compared to the microsatellite profile of the mother plants.

### Statistical analysis

The data were analyzed using the Statistical Analysis System (SAS) version eight statistical package (SAS Institute Inc., Cary, NC, USA). A two-way factorial analysis was conducted to study the effects of the medium and genotype on the percentages of embryogenic explants (E) and embryogenic explants produced via direct embryogenesis (DE) after two, three, and 4 months of culture, as well as the plant embryo conversion (EC) - measured as the percentage of embryos with normal cotyledons and apex- and plant development, measured as a shoot development index (DI) and a rooting index (RI), after 20 and 40 days of culture. The interaction of both effects was also included - to analyze the number of embryos per explant (NE) for each cultivar after two and 3 months of culture, and its average. Differences for all traits among the 14 genotypes were evaluated by analysis of variance (ANOVA) testing, except for variety Mencía which was not included for the plant embryo conversion and plant development measurements due to contamination of some tubes. The significance of the differences was determined by a least significant difference (LSD).

## Additional files

Additional file 1: Table S1. Plant development measured using a shoot development index (DI) and a rooting index (RI), after 20 and 40 day of culture. As starting explants, 20 somatic embryos from direct embryogenesis events were sown in individual tubes. (DOCX 13 kb)

Additional file 2: Table S2. Virus status and microsatellites (SSRs) profile of mother plants and plantlets from somatic embryos of cultivars Tempranillo and Pinot Blanc plants. (DOCX 15 kb)

Additional file 3: Table S3. SSR profiles of the varieties Cabernet Franc, Godello, Merlot and Valencí Blanc and those regenerated through somatic embryogenesis. (DOCX 17 kb)

Additional file 4: Table S4. Sequences of the forward and reverse primers and, probes used for TaqMan® RT-PCR. (DOCX 12 kb)

## Abbreviations

2,4-D: Dichlorophenoxiacetic acid
6-BA: Benzylaminopurine
AC: Activated charcoal
ArMV: Arabis mosaic virus
DE: Direct embtyogenesis
DI: Development index
E: Percentage of somatic embryogenic explants
EC: Embryo conversion
EIM2: Embryogenesis induction medium 2
G: Seed germination
GFkV: Grapevine fleck virus.
GFLV: Grapevine fanleaf virus
GLRaV: Grapevine leafroll associated viruses
GLRaV-1: Grapevine leafroll associated virus 1
GLRaV-3: Grapevine leafroll associated virus 3
MS: Murashige and Skoog basal medium
NE: Number of embryos per responding explant
PVP-10: Polyvinylpyrrolidone-10
RI: Rooting index
RW: Rugose wood
SE: Somatic embryogenesis
TDZ: Thidiazuron
