# Supplementary material for: Somatic embryogenesis from seeds in a broad range of Vitis vinifera L. varieties: rescue of true-to-type virus-free plants
Source: BMC Plant Biol. 2017 Nov 29;17:226. doi: 10.1186/s12870-017-1159-3 (PMC5706158; doi:10.1186/s12870-017-1159-3)
Supplement: Supplementary file 1 — Plant development measured using a shoot development index (DI) and a rooting index (RI), after 20 and 40 day of culture. As starting explants, 20 somatic embryos from direct embryogenesis events were sown in individual tubes. (DOCX 13 kb) [file 12870_2017_1159_MOESM1_ESM.docx]

**Table S1. Plant development measured using a shoot development index (DI) and a rooting index (RI), after 20 and 40 day of culture. As starting explants, 20 somatic embryos from direct embryogenesis events were sown in individual tubes.**

| **Cultivar** | **DI^a^** | **RI^b^** | **DI^a^** | **RI^b^** |
| --- | --- | --- | --- | --- |
|  | **20 days** | | **40 days** | |
| Airén | 0.22 bc | 2.14 bc | 1.23 de | 2.74 a |
| Cabernet Franc | 0.56 bc | 1.79 bc | 3.75 abcd | 2.38 a |
| Cabernet Sauvignon | 0.00 c | 1.76 c | 0.39 e | 2.08 a |
| Merlot | 0.30 bc | 1.60 c | 3.85 abc | 2.75 a |
| Monastrell | 0.41 bc | 1.71 c | 1.73 cde | 2.18 a |
| Petit Verdot | 0.37 bc | 1.79 bc | 2.79 bcde | 2.17 a |
| Pinot Blanc | 0.67 bc | 2.32 ab | 1.65 cde | 2.86 a |
| Pinot Gris | 1.04 bc | 1.72 c | 2.96 bcd | 2.04 a |
| Pinot Meunier | 3.84 a | 1.73 c | 5.66 a | 2.20 a |
| Pinot Noir | 0.57 bc | 1.83 bc | 3.11 bcd | 2.26 a |
| Syrah | 0.44 bc | 1.89 bc | 1.29 de | 2.31 a |
| Tempranillo | 1.43 b | 2.31 ab | 5.29 ab | 2.96 a |
| Verdil | 0.23 bc | 2.69 a | 2.72 cde | 2.93 a |

^a^DI was calculated by multiplying the percentage of plantlets with leaves (expressed as a decimal) by the mean number of leaves per plantlet with leaves.

^b^RI is a qualitative index (RI: 1-3, 1: small roots; 2: main root 1.0-1.5 cm or presence of some secondary roots, 3: main root > 1.5 cm and/or with many secondary roots) used for scoring the rooting.

Different lower case letters within a column indicate significantly different values (P value<0.05).
