# Supplementary material for: Somatic embryogenesis from seeds in a broad range of Vitis vinifera L. varieties: rescue of true-to-type virus-free plants
Source: BMC Plant Biol. 2017 Nov 29;17:226. doi: 10.1186/s12870-017-1159-3 (PMC5706158; doi:10.1186/s12870-017-1159-3)
Supplement: Supplementary file 2 — Virus status and microsatellites (SSRs) profile of mother plants and plantlets from somatic embryos of cultivars Tempranillo and Pinot Blanc plants. (DOCX 15 kb) [file 12870_2017_1159_MOESM2_ESM.docx]

**Table S2. Virus status and microsatellites (SSRs) profile of mother plants and plantlets from somatic embryos of cultivars Tempranillo and Pinot Blanc plants.**

|  |  | **VVS2** | | **VVMD5** | | **VVMD7** | | **VVMD27** | | **VrZAG62** | | **VrZAG79** | |
| --- | --- | --- | --- | --- | --- | --- | --- | --- | --- | --- | --- | --- | --- |
| **Cultivar** | **Virus status of plants** | **Allele 1** | **Allele 2** | **Allele 1** | **Allele 2** | **Allele 1** | **Allele 2** | **Allele 1** | **Allele 2** | **Allele 1** | **Allele 2** | **Allele 1** | **Allele 2** |
| **Tempranillo (T) mother plant** | **GFLV** | **143** | **145** | **238** | **238** | **239** | **253** | **184** | **184** | **196** | **200** | **247** | **251** |
| SE-T-s2.6 | Free | 143 | 145 | 238 | 238 | 239 | 253 | 184 | 184 | 200 | 200 | 247 | 251 |
| SE-T-s2.9 | GFLV | 143 | 145 | 238 | 238 | 253 | 253 | 184 | 184 | 200 | 200 | 247 | 247 |
| SE-T-s2.28 | GFLV | 143 | 145 | 238 | 238 | 239 | 253 | 184 | 184 | 200 | 200 | 247 | 251 |
| SE-T-s2.31 | Free | 143 | 145 | 238 | 238 | 253 | 253 | 184 | 184 | 200 | 200 | 247 | 247 |
| **SE-T-s2.65** | Free | **143** | **145** | **238** | **238** | **239** | **253** | **184** | **184** | **196** | **200** | **247** | **251** |
| SE-T-s2.68 | Free | 143 | 145 | 238 | 238 | 253 | 253 | 184 | 184 | 200 | 200 | 247 | 247 |
| SE-T-s2.89 | GFLV | 143 | 145 | 238 | 238 | 253 | 253 | 184 | 184 | 200 | 200 | 247 | 247 |
| SE-T-s2.92 | Free | 143 | 145 | 238 | 238 | 253 | 253 | 184 | 184 | 200 | 200 | 247 | 247 |
| SE-T-s2.101 | GFLV | 145 | 145 | 238 | 238 | 239 | 239 | 184 | 186 | 196 | 200 | 247 | 247 |
| SE-T-s2.104 | Free | 143 | 145 | 238 | 238 | 253 | 253 | 184 | 184 | 200 | 200 | 247 | 247 |
| SE-T-s2.107 | Free | 145 | 145 | 238 | 238 | 239 | 239 | 184 | 186 | 196 | 200 | 247 | 247 |
| SE-T-s2.125 | Free | 143 | 145 | 238 | 238 | 253 | 253 | 184 | 184 | 200 | 200 | 247 | 247 |
| SE-T-s2.128 | GFLV | 143 | 145 | 238 | 238 | 253 | 253 | 184 | 184 | 200 | 200 | 247 | 247 |
| SE-T-s2.154 | Free | 143 | 145 | 238 | 238 | 239 | 239 | 184 | 184 | 196 | 196 | 247 | 247 |
| **SE-T-s3.40** | Free | **143** | **145** | **238** | **238** | **239** | **253** | **184** | **184** | **196** | **200** | **247** | **251** |
| SE-T-s3.41 | Free | 145 | 145 | 238 | 238 | 239 | 239 | 184 | 186 | 196 | 200 | 247 | 247 |
| **Pinot Blanc mother plant** | **GFLV and GFkV** | **137** | **151** | **230** | **240** | **239** | **243** | **186** | **190** | **188** | **194** | **239** | **245** |
| SE-PB-s2.61 | Free | 151 | 151 | 240 | 240 | 239 | 243 | 186 | 186 | 194 | 194 | 245 | 245 |
| SE-PB-s2.141 | GFLV | 151 | 151 | 240 | 240 | 239 | 243 | 186 | 186 | 194 | 194 | 245 | 245 |
| SE-PB-s3.58 | Free | 137 | 137 | 230 | 230 | 239 | 239 | 190 | 190 | 194 | 194 | 239 | 239 |
| SE-PB-s3.59 | Free | 137 | 151 | 240 | 240 | 239 | 243 | 186 | 190 | 188 | 194 | 239 | 245 |
| **SE-PB-s3.60** | Free | **137** | **151** | **230** | **240** | **239** | **243** | **186** | **190** | **188** | **194** | **239** | **245** |
| SE-PB-s3.152 | Free | 137 | 151 | 230 | 230 | 239 | 243 | 186 | 190 | 188 | 194 | 239 | 245 |
| SE-PB-s3.153 | Free | 137 | 151 | 230 | 230 | 239 | 243 | 186 | 190 | 188 | 194 | 239 | 245 |
| SE-PB-s4.56 | Free | 137 | 151 | 230 | 230 | 239 | 243 | 186 | 190 | 188 | 194 | 239 | 245 |

GFLV: Grapevine fanleaf virus, GFkV: Grapevine fleck virus. In bold mother plants and SE plants with the same profile to the mother plant.
