# Supplementary material for: Somatic embryogenesis from seeds in a broad range of Vitis vinifera L. varieties: rescue of true-to-type virus-free plants
Source: BMC Plant Biol. 2017 Nov 29;17:226. doi: 10.1186/s12870-017-1159-3 (PMC5706158; doi:10.1186/s12870-017-1159-3)
Supplement: Supplementary file 3 — SSR profiles of the varieties Cabernet Franc, Godello, Merlot and Valencí Blanc and those regenerated through somatic embryogenesis. (DOCX 17 kb) [file 12870_2017_1159_MOESM3_ESM.docx]

**Table S3. SSR profiles of the varieties Cabernet Franc, Godello, Merlot and Valencí Blanc and those regenerated through somatic embryogenesis**

| **Cultivar** | **VVMD7** | | **VVMD5** | | **VVS2** | | **VrZAG79** | | **VrZAG62** | | **VVMD7** | | **VVMD25** | | **VVMD28** | | **VVMD32** | |
| --- | --- | --- | --- | --- | --- | --- | --- | --- | --- | --- | --- | --- | --- | --- | --- | --- | --- | --- |
| **Cabernet Franc** | 179 | 187 | 223 | 237 | 140 | 148 | 246 | 258 | 195 | 205 | 237 | 261 | 239 | 255 | 227 | 235 | 238 | 256 |
| SE CF-S1.107 | 179 | 179 | 223 | 237 | 140 | 148 | 246 | 258 | 195 | 205 | 237 | 261 |  |  |  |  |  |  |
| **SE-CF-S1.150** | **179** | **187** | **223** | **237** | **140** | **148** | **246** | **258** | **195** | **205** | **237** | **261** | **239** | **255** | **227** | **235** | **238** | **256** |
| SE-CF-S1.157 | 179 | 179 | 223 | 237 | 140 | 148 | 246 | 258 | 195 | 205 | 237 | 261 |  |  |  |  |  |  |
| SE-CF-S1.158 | 179 | 187 | 223 | 237 | 140 | 148 | 246 | 258 | 205 | 205 | 237 | 261 |  |  |  |  |  |  |
| SE-CF-S2.37 | 179 | 179 | 223 | 237 | 140 | 148 | 246 | 258 | 205 | 205 | 237 | 261 |  |  |  |  |  |  |
| SE-CF-S2.40 | 179 | 187 | 223 | 223 | 140 | 148 | 246 | 258 | 195 | 205 | 237 | 261 |  |  |  |  |  |  |
| SE-CF-S2.59 | 179 | 187 | 223 | 223 | 140 | 148 | 246 | 258 | 195 | 205 | 237 | 261 |  |  |  |  |  |  |
| SE-CF-S2.77 | 187 | 187 | 223 | 237 | 140 | 148 | 246 | 258 | 195 | 205 | 237 | 261 |  |  |  |  |  |  |
| SE-CF-S2.80 | 179 | 187 | 223 | 237 | 140 | 148 | 246 | 258 | 195 | 205 | 237 | 237 |  |  |  |  |  |  |
| SE-CF-S2.110 | 179 | 187 | 223 | 223 | 140 | 148 | 246 | 258 | 195 | 205 | 237 | 261 |  |  |  |  |  |  |
| SE-CF-S2.122 | 179 | 187 | 223 | 237 | 140 | 148 | 246 | 258 | 195 | 205 | 237 | 237 |  |  |  |  |  |  |
| SE-CF-S2.157 | 179 | 187 | 223 | 237 | 140 | 148 | 246 | 258 | 195 | 205 | 237 | 261 |  |  |  |  |  |  |
| SE-CF-S2.150 | 179 | 187 | 223 | 237 | 140 | 148 | 246 | 258 | 205 | 205 | 237 | 261 |  |  |  |  |  |  |
| SE-CF-S3.101 | 179 | 187 | 223 | 237 | 140 | 148 | 246 | 258 | 205 | 205 | 237 | 261 |  |  |  |  |  |  |
| SE-CF-S3.102 | 179 | 187 | 223 | 223 | 140 | 148 | 246 | 258 | 205 | 205 | 237 | 261 |  |  |  |  |  |  |
|  |  |  |  |  |  |  |  |  |  |  |  |  |  |  |  |  |  |  |
| **Godello** | **183** | **187** | **223** | **235** | **154** | **160** | **250** | **250** | **187** | **187** | **237** | **237** | 249 | 249 | 235 | 257 | 250 | 270 |
| SE-G-S1.59 | 187 | 187 | 223 | 235 | 154 | 160 | 250 | 250 | 187 | 187 | 237 | 237 |  |  |  |  |  |  |
| SE-G-S1.101 | 187 | 187 | 223 | 235 | 154 | 160 | 250 | 250 | 187 | 187 | 237 | 237 |  |  |  |  |  |  |
| SE-G-S1.136 | 187 | 187 | 223 | 235 | 154 | 160 | 250 | 250 | 187 | 187 | 237 | 237 |  |  |  |  |  |  |
| **SE-G-S2.30** | **183** | **187** | **223** | **235** | **154** | **160** | **250** | **250** | **187** | **187** | **237** | **237** | **249** | **249** | **235** | **257** | **250** | **270** |
| SE-G-S2.84 | 183 | 187 | 235 | 235 | 154 | 160 | 250 | 250 | 187 | 187 | 237 | 237 |  |  |  |  |  |  |
| SE-G-S2.123 | 183 | 187 | 223 | 235 | 154 | 160 | 250 | 250 | 187 | 201 | 237 | 237 |  |  |  |  |  |  |
| SE-G-S2.145 | 183 | 187 | 223 | 223 | 154 | 160 | 250 | 250 | 187 | 187 | 237 | 237 |  |  |  |  |  |  |
| SE-G-S3.61 | 183 | 187 | 223 | 223 | 154 | 160 | 250 | 250 | 187 | 201 | 237 | 237 |  |  |  |  |  |  |
| SE-G-S3.62 | 183 | 187 | 223 | 223 | 154 | 160 | 250 | 250 | 187 | 187 | 237 | 237 |  |  |  |  |  |  |
| SE-G-S3.115 | 187 | 187 | 223 | 235 | 154 | 160 | 250 | 250 | 187 | 187 | 237 | 237 |  |  |  |  |  |  |
| SE-G-S3.136 | 187 | 187 | 235 | 235 | 154 | 160 | 250 | 250 | 187 | 187 | 237 | 237 |  |  |  |  |  |  |
|  |  |  |  |  |  |  |  |  |  |  |  |  |  |  |  |  |  |  |
| **Merlot** | **185** | **189** | **223** | **233** | **140** | **153** | **258** | **258** | **195** | **195** | **237** | **245** | 239 | 249 | 227 | 233 | 238 | 238 |
| SE-M-S1.37 | 185 | 189 | 223 | 223 | 140 | 153 | 258 | 258 | 195 | 195 | 237 | 245 |  |  |  |  |  |  |
| SE-M-S2.7 | 185 | 189 | 233 | 233 | 140 | 153 | 258 | 258 | 195 | 195 | 237 | 245 |  |  |  |  |  |  |
| SE-M-S2.24 | 185 | 189 | 223 | 233 | 140 | 153 | 258 | 258 | 195 | 201 | 237 | 245 |  |  |  |  |  |  |
| SE-M-S2.46 | 185 | 189 | 223 | 233 | 140 | 153 | 258 | 258 | 195 | 195 | 237 | 237 |  |  |  |  |  |  |
| SE-M-S2.137 | 185 | 189 | 223 | 233 | 140 | 153 | 258 | 258 | 195 | 195 | 237 | 237 |  |  |  |  |  |  |
| **SE-M-S3.37** | **185** | **189** | **223** | **233** | **140** | **153** | **258** | **258** | **195** | **195** | **237** | **245** | **239** | **249** | **227** | **233** | **238** | **238** |
| SE-M-S3.38 | 185 | 189 | 223 | 233 | 140 | 153 | 258 | 258 | 195 | 201 | 237 | 245 |  |  |  |  |  |  |
| SE-M-S3.39 | 185 | 185 | 223 | 233 | 140 | 153 | 258 | 258 | 195 | 195 | 237 | 245 |  |  |  |  |  |  |
|  |  |  |  |  |  |  |  |  |  |  |  |  |  |  |  |  |  |  |
| **Valencí Blanc** | **179** | **187** | **233** | **237** | **136** | **145** | **243** | **247** | **205** | **205** | **241** | **247** | **255** | **255** | **243** | **257** | **254** | **254** |
| **SE-VB**-**L 1.10** | **179** | **187** | **233** | **237** | **136** | **145** | **243** | **247** | **205** | **205** | **241** | **247** | **255** | **255** | **243** | **257** | **254** | **254** |
| SE-VB-L 2.1 | 179 | 179 | 233 | 237 | 145 | 145 | 243 | 247 | 205 | 205 | 241 | 247 |  |  |  |  |  |  |
| SE-VB-L 2.6 | 179 | 179 | 233 | 237 | 136 | 145 | 243 | 247 | 205 | 205 | 241 | 247 |  |  |  |  |  |  |
| SE-VB-L 3.1 | 179 | 187 | 237 | 237 | 136 | 145 | 243 | 247 | 205 | 205 | 241 | 247 |  |  |  |  |  |  |
| SE-VB-L 3.2 | 179 | 179 | 233 | 237 | 136 | 145 | 243 | 247 | 205 | 205 | 241 | 247 |  |  |  |  |  |  |
| SE-VB-L 4.4 | 179 | 187 | 233 | 237 | 145 | 145 | 243 | 247 | 205 | 205 | 241 | 241 |  |  |  |  |  |  |
| SE-VB-L 5.1 | 179 | 187 | 233 | 237 | 136 | 145 | 243 | 247 | 205 | 205 | 241 | 241 |  |  |  |  |  |  |
| SE-VB-L 7.1 | 179 | 187 | 233 | 233 | 136 | 145 | 243 | 247 | 205 | 205 | 241 | 247 |  |  |  |  |  |  |
| SE-VB-L 10.1 | 179 | 187 | 233 | 233 | 136 | 145 | 243 | 247 | 205 | 205 | 241 | 247 |  |  |  |  |  |  |
| SE-VB-L 10.2 | 179 | 179 | 233 | 237 | 136 | 145 | 243 | 247 | 205 | 205 | 241 | 247 |  |  |  |  |  |  |
