# Supplementary material for: Somatic embryogenesis from seeds in a broad range of Vitis vinifera L. varieties: rescue of true-to-type virus-free plants
Source: BMC Plant Biol. 2017 Nov 29;17:226. doi: 10.1186/s12870-017-1159-3 (PMC5706158; doi:10.1186/s12870-017-1159-3)
Supplement: Supplementary file 4 — Sequences of the forward and reverse primers and, probes used for TaqMan® RT-PCR. (DOCX 12 kb) [file 12870_2017_1159_MOESM4_ESM.docx]

**Table S4: Sequences of the forward and reverse primers and, probes used for TaqMan® RT-PCR.**

| **Virus** | **Primer forward** | **Primer reverse** | **Probe** |
| --- | --- | --- | --- |
| ArMV | 5′-TAG CCC TTG TAC TTA TGG CA-3′ | 5′-TAT TTA AAC AGT TGA TTC CA-3′ | 5′-TTG TTA GTG AAT GGA ACG GGG TCA-3′ |
| GFLV | 5′-GGGACCACTATGGATGGAATGA-3′ | 5′-TTCGGTGATATGGAGAGCGAAT-3′ | 5′-AGT GGA ACG GGA CCA C-3′ |
| GFkV | 5′-CGAGAACTCTCTTTTCACCTC-3′ | 5′-CCGGCGTGGATGTAGAG-3′ | 5′-ACCCTCGCCCTCATGCA-3′ |
| GLRaV-1 | 5′-ACCTGGTTGAACGAGATCGCTT-3′ | 5′-GTAAACGGGTGTTCTTCAATTCTCT-3′ | 5′-ACGAGATATCTGTGGACGGA-3′ |
| GLRaV-3 | 5′-AAGTGCTCTAGTTAAGGTCAGGAGTGA-3′ | 5′-GTATTGGACTACCTTTCGGGAAAAT-3′ | 5′-CAGGTAATAGCGGACTGAGACTGGTGGACA-3′ |
